# Causal relationships between human blood metabolites and intracranial aneurysm and aneurysmal subarachnoid hemorrhage: a Mendelian randomization study

**DOI:** 10.3389/fneur.2023.1268138

**Published:** 2023-12-14

**Authors:** Jia Jiang, Siming Gui, Dachao Wei, Xiheng Chen, Yudi Tang, Jian Lv, Wei You, Ting Chen, Shu Yang, Huijian Ge, Youxiang Li

**Affiliations:** ^1^Department of Neurosurgery, Beijing Neurosurgical Institute and Beijing Tiantan Hospital, Capital Medical University, Beijing, China; ^2^Department of Neurosurgery, Beijing Chaoyang Hospital, Capital Medical University, Beijing, China; ^3^School of Biomedical Engineering, Capital Medical University, Beijing, China

**Keywords:** intracranial aneurysm, blood metabolites, Mendelian randomization, risk factors, single-nucleotide polymorphisms

## Abstract

**Objective:**

The aim of this study was to assess the causal relationships between blood metabolites and intracranial aneurysm, aneurysmal subarachnoid hemorrhage, and unruptured intracranial aneurysm.

**Methods:**

Our exposure sample consisted of 7,824 individuals from a genome-wide association study of human blood metabolites. Our outcome sample consisted of 79,429 individuals (7,495 cases and 71,934 controls) from the International Stroke Genetics Consortium, which conducted a genome-wide association study of intracranial aneurysm, aneurysmal subarachnoid hemorrhage, and unruptured intracranial aneurysm. We identified blood metabolites with a potential causal effect on intracranial aneurysms and conducted sensitivity analyses to validate our findings.

**Results:**

After rigorous screening and Mendelian randomization tests, we found four, two, and three serum metabolites causally associated with intracranial aneurysm, aneurysmal subarachnoid hemorrhage, and unruptured intracranial aneurysm, respectively (all *P* < 0.05). Sensitivity analyses confirmed the robustness of these associations.

**Conclusions:**

Our Mendelian randomization analysis demonstrated causal relationships between human blood metabolites and intracranial aneurysm, aneurysmal subarachnoid hemorrhage, and unruptured intracranial aneurysm. Further research is required to explore the potential of targeting these metabolites in the management of intracranial aneurysm.

## Introduction

The prevalence of intracranial aneurysms (IAs) in the general population worldwide is approximately 3%−5% (1). IAs are characterized by localized deterioration of the arterial wall structure, loss of the internal elastic plate, and disruption of the arterial media (2). The most severe complication of IAs is rupture, accounting for ~85% of spontaneous subarachnoid hemorrhages (SAHs) (3). Aneurysmal SAH (aSAH) from an IA typically leads to a poor prognosis, with high rates of disability and mortality (4, 5). Despite the recognized contribution of genetic and environmental factors and their complex interactions in IAs, a better understanding of the pathogenesis of IAs is required.

Previous investigations have proposed that metabolites function as intermediary components, facilitating exploration of the connection between genetic variation and metabolites (6). In an observational study, Duan et al. found a significantly disrupted metabolic profile in the IA population compared with that in the normal population. Serum adenosine was significantly decreased in patients with unruptured IA (UIA) compared with that in the normal population, whereas serum levels of various glycine-conjugated secondary bile acids were decreased in patients with aSAH (7). Cotinine is a major metabolite of nicotine, and Missori et al. found that in a population of smokers with IA, aneurysms were more likely to rupture in people with high rather than low cotinine levels (8). Therefore, establishing a causal relationship between these two factors is essential for developing effective metabolite-targeted interventions to prevent and manage IAs in clinical practice.

Mendelian randomization (MR) is a statistical technique that enables the inference of causality between exposure and outcome using genetic variants associated with exposure as instrumental variables (IVs) (9–11). Whether an instrumental variable is valid depends on the fulfillment of three basic assumptions: they are required to demonstrate a clear association with the particular risk factor of interest (the relevance assumption); they must have no causal determinants in common with the outcome under investigation (the independence assumption); and they must not have any direct effect on the outcome variable, except through their effect on intermediate risk factors (the exclusion restriction assumption) (10). Compared with observational studies, MR studies are less susceptible to confounding because genetic variants are determined and randomly assigned. Thus, this design, similar to previous MR studies, helps to identify causal associations of a large number of serum metabolites with IAs (12, 13). Large-scale genome-wide association studies (GWASs) have provided promising results for MR analyses of various diseases, including those related to human blood metabolites (14). However, no MR study has been conducted to examine the potential causal relationship between blood metabolites and IAs.

In the present study, we aimed to investigate the possible causal relationships between blood metabolites and IAs through a comprehensive two-sample MR analysis.

## Methods

### Study design and data sources

**Figure 1:**
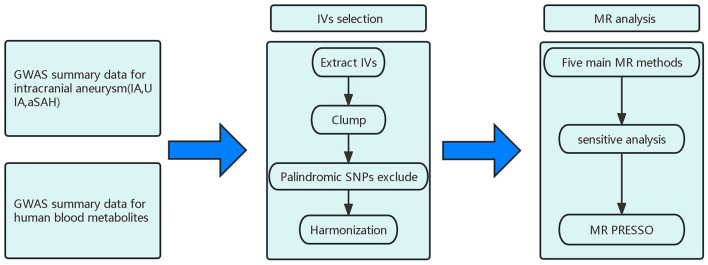
Design and workflow of this study; IA, Intracranial aneurysm; UIA, Unruptured intracranial aneurysm; aSAH, aneurysmal Subarachnoid hemorrhage; IV, Instrumental variable; SNP, Single-nucleotide polymorphism; GWAS, Genome-wide association study; MR, Mendelian randomization.

The flow of this study is shown in Figure 1. This study used summary-level data from publicly available GWASs. The GWAS examining human blood metabolites was conducted by Shin and colleagues, and the GWAS investigating IAs was performed by the Intracranial Aneurysm working group of the International Stroke Genetics Consortium (14, 15). The GWAS studies had ethical approval and participant consent, and de-identified aggregate data were made available for analysis. This MR study was conducted based on strengthening the reporting of observational studies in epidemiology (STROBE-MR) guidelines (Supplementary Table 1) (16).

### Blood metabolite samples

The blood metabolite summary statistics were sourced from an extensive investigation conducted by Shin and colleagues, which stands as one of the most comprehensive studies in the field (14). A sample size of 7,824 adults from Europe was collected for the GWAS analysis, with over 2.1 million single-nucleotide polymorphisms (SNPs) analyzed from this population. Following rigorous quality control measures, a total of 486 metabolites were deemed suitable for the GWAS analysis. These metabolites were further classified into 309 known metabolites and 177 unknown metabolites. The 309 known metabolites were categorized into eight distinct biochemical groups, namely amino acids, peptides, lipids, cofactors and vitamins, carbohydrates, energy-related molecules, nucleotides, and exotic organisms.

### IA sample

Summary data for IA patients were collected from a GWAS of 23 cohorts with 79,429 individuals, including 7,495 cases and 71,934 controls. Among the total cases, 69% had a ruptured IA, 28% had a UIA, and 3.8% had an unknown rupture status (15). The present study analyzed three summary datasets and applied MR techniques. The three datasets were as follows: a GWAS of IA cases (ruptured, unruptured, and unclear) (*n* = 7,495), a GWAS of UIA cases (*n* = 2,070), and a GWAS of aSAH cases (*n* = 5,140); each dataset was compared with that of the control group (*n* = 71,934). All participants in the study were of European descent.

### Selection of IVs

Genome-wide statistically significant SNPs associated with blood metabolites were used as IVs, and the genetic instruments used a value of P < 5 × 10^−6^. Several quality control steps were performed to ensure eligible IV selection, including eliminating SNPs with inconsistent alleles between the exposure and outcome samples (i.e., A/G vs. A/C), retaining only independent SNPs from each blood metabolite, setting the linkage disequilibrium threshold for clumping to *r*^2^ < 0.01, and using a clumping window size of 500 kb while also excluding alleles with palindromic A/T or G/C combinations. Finally, to evaluate the robustness of the selected SNPs, we evaluated the SNP power using F statistics (F = beta^2^/se^2^) and the variance explained by IVs [R^2^ = 2 × MAF × (1-MAF) × (beta/sd)^2^] (17, 18). A value of F ≥ 10 indicates a lack of weak instrument bias, and IVs with F statistics <10 were excluded as weak (19). The objective of this analysis was to ensure the validity of the IVs employed in the study.

### Statistical analysis

This study used the following five methods of MR analysis to examine the association between blood metabolites and IAs: inverse-variance weighted (IVW), weighted median, MR-Egger, weighted mode, and simple mode (20). To address the issue of multiple testing, we employed the Bonferroni correction and set the significance threshold to P < 0.05/n (where n is the number of metabolites finally included in the MR analysis) (21). Suggestive associations were considered when the *P* value for the uncorrected IVW method was <0.05. When the *P* value after Bonferroni correction was <0.0001 (0.05 divided by 486), the serum metabolite was considered to be significantly associated with IAs. We performed sensitivity analyses only for metabolites with *P* < 0.05 for both the IVW method and weighted median method results in the MR analysis. MR-Egger regression analysis was conducted to evaluate potential directional pleiotropy. We used the MR Pleiotropy RESidual Sum and Outlier (MR-PRESSO) global test to detect overall horizontal pleiotropy and eliminated any pleiotropic effects by removing outliers (22). We employed Cochran's Q test to assess heterogeneity among SNPs associated with each blood metabolite. We also conducted a leave-one-out analysis to determine whether a single SNP was responsible for significant results. All statistical analyses were performed using R (version 4.2.2), and the MR and sensitivity analyses were conducted using the two-sample MR and MR-PRESSO R packages.

## Results

### Selection of IVs

After a series of quality controls, we identified 4,065 independent SNPs associated with 486 blood metabolites. Specific information on all SNPs used as genetic IVs can be found in Supplementary Table 2. The F-values calculated for all IVs were >10, indicating that there were no weak IVs selected from the 486 metabolites.

### Effects of genetically determined metabolites on IA

**Figure 2:**
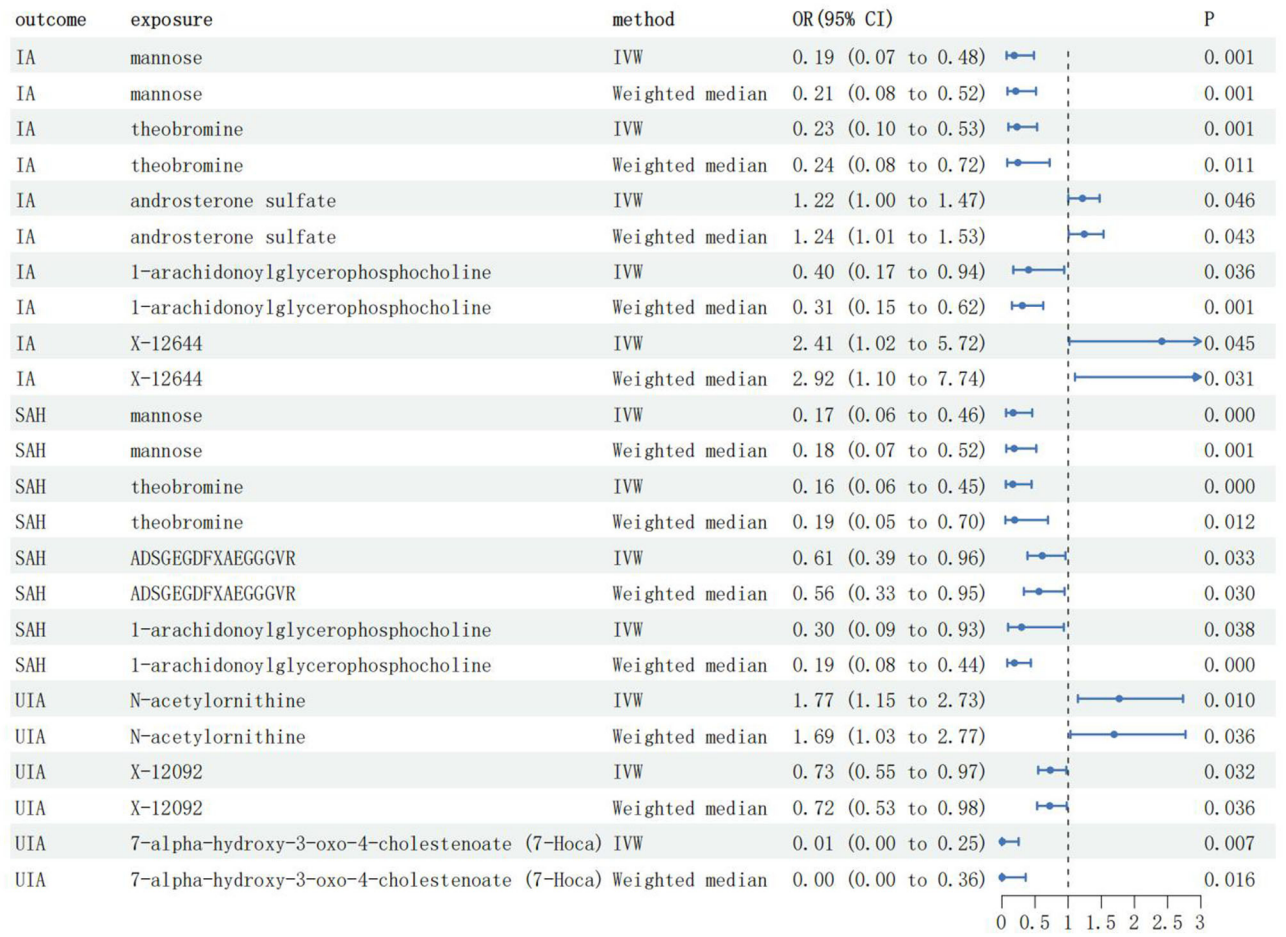
The main results of MR analysis; IA, Intracranial aneurysm; UIA, Unruptured intracranial aneurysm; aSAH, aneurysmal Subarachnoid hemorrhage; IVW, Inverse-variance weighted; OR, Odds ratio; CI, Confidence interval.

To ensure the robustness of the results, only results with a *P* < 0.05 for both the IVW method and the weighted median method were included in the final study results (Supplementary Table 3). As shown in Figure 2, we detected five IA-associated, four aSAH-associated, and three UIA-associated metabolites. Among the blood metabolites associated with IA, three metabolites, mannose [odds ratio (OR) = 0.19, 95% confidence interval (CI): 0.07–0.48, P_IVW_=0.001], theobromine (OR = 0.23, 95% CI: 0.10–0.53, P_IVW_ = 0.001), and 1-arachidonoylglycerophosphocholine (OR = 0.40, 95% CI: 0.17–0.94, P_IVW_ = 0.036), were suggestive associated with a reduced risk of IA, and two metabolites, androsterone sulfate (OR = 1.22, 95% CI: 1.00–1.47, P_IVW_ = 0.046) and X-12644 (OR = 2.41, 95% CI: 1.02–5.72, P_IVW_ = 0.045), were suggestive associated with an increased risk of IA. Specifically, each standard deviation (SD) increase in levels of mannose, theobromine, and 1-arachidonoylglycerophosphocholine was suggestive of an associated reduction in the risk of IA by 81%, 77%, and 60%, respectively. Each SD increase in androsterone sulfate and X-12644 levels was suggestive of an associated increase in the risk of IA by 22% and 141%, respectively.

### Effects of genetically determined metabolites on aSAH

Among the blood metabolites associated with aSAH, four specific metabolites exhibited notable findings. Mannose (OR = 0.17, 95% CI: 0.06–0.46, P_IVW_ < 0.001), theobromine (OR = 0.16, 95% CI: 0.06–0.45, P_IVW_ < 0.001), ADSGEGDFXAEGGGVR (OR = 0.61, 95% CI: 0.39–0.96, P_IVW_ = 0.033), and 1-arachidonoylglycerophosphocholine (OR = 0.30, 95% CI: 0.09–0.93, P_IVW_ = 0.038) were suggestive associated with a reduced risk of aSAH. Specifically, SD increases in mannose, theobromine, ADSGEGDFXAEGGGVR, and 1-arachidonoylglycerophosphocholine levels were suggestive of an associated reduction in the aSAH risk by 83%, 84%, 39%, and 70%, respectively.

### Effects of genetically determined metabolites on UIA

Among the blood metabolites associated with UIA, two specific metabolites, X-12092 (OR = 0.73, 95% CI: 0.55–0.97, P_IVW_ = 0.032) and 7-alpha-hydroxy-3-oxo-4-cholestenoate (OR = 0.02, 95% CI: 0.01–0.25, P_IVW_ = 0.007), were suggestive associated with a reduced risk of IA. Conversely, N-acetylornithine (OR = 1.77, 95% CI: 1.15–2.73, P_IVW_ = 0.010) was suggestive associated with an increased risk of UIA. Specifically, SD increases in X-12092 and 7-alpha-hydroxy-3-oxo-4-cholestenoate levels were associated with a 27% and 98% reduction in UIA risk, respectively. Each SD increase in N-acetylornithine levels suggested an association with a 77% increase in RIA risk.

### Sensitivity analysis

Cochran's Q test showed no heterogeneity between the relevant SNPs for each serum metabolite (all *P* > 0.05, Table 1). Additionally, the MR-Egger regression analysis did not reveal any potential for directional pleiotropy, except for the effect of 1-arachidonoylglycerophosphocholine on aSAH (*P* = 0.028, Table 2). Because there was not a sufficient number of IVs to assess theobromine, it was not possible to test for potential horizontal pleiotropy using MR-PRESSO and global testing methods, and the remaining metabolites showed no horizontal pleiotropy (*P* > 0.05, Supplementary Table 4). We performed leave-one-out analyses and identified strong effects of the SNP rs174535 on the causal associations of 1-arachidonoylglycerophosphocholine with IA and aSAH. The leave-one-out analysis indicated that other metabolites did not differ significantly in their estimated causal effects on IA, aSAH, and UIA. This finding suggests that no single independent variable was driving the identified causal associations (Supplementary Figure S1). Therefore, we excluded the MR analyses results of 1-arachidonoylglycerophosphocholine on IA and aSAH and those of ADSGEGDFXAEGGGVR on aSAH.

**Table 1:** The heterogeneity results from the Cochran's Q test.

**Outcome**	**Exposure**	**Q**	***P*-value**
IA	Mannose	3.763369	0.2881758
IA	Theobromine	0.14660755	0.9293185
IA	Androsterone sulfate	5.952839	0.5452668
IA	1-arachidonoylglycerophosphocholine	12.187059	0.09457271
IA	X-12644	20.01434	0.09485329
SAH	Mannose	2.365225	0.5001408
SAH	Theobromine	1.0392924	0.5947309
SAH	ADSGEGDFXAEGGGVR	0.889303	0.9260904
SAH	1-arachidonoylglycerophosphocholine	8.72387	0.05926614
UIA	N-acetylornithine	9.794632	0.458692
UIA	X-12092	6.57921	0.6808369
UIA	7-alpha-hydroxy-3-oxo-4-cholestenoate (7-Hoca)	2.0152106	0.5692557

**Table 2:** Directional pleiotropy results from Egger intercept analysis.

**Outcome**	**Exposure**	**Egger_intercept**	**SE**	***P*-value**
IA	Mannose	−0.01345948	0.04362969	0.7868738
IA	Theobromine	−0.05128786	0.1692686	0.812704
IA	Androsterone sulfate	−0.01119303	0.01305627	0.4241972
IA	1-arachidonoylglycerophosphocholine	0.02927189	0.01528644	0.104
IA	X-12644	−0.02145262	0.02151067	0.3383041
SAH	Mannose	−0.01381247	0.03974681	0.7613712
SAH	Theobromine	−0.07714656	0.1977636	0.1977636
SAH	ADSGEGDFXAEGGGVR	−0.006628132	0.02819927	0.02819927
SAH	1-arachidonoylglycerophosphocholine	0.03894936	0.02141559	0.1188277
UIA	N-acetylornithine	0.01112292	0.02159327	0.6188877
UIA	X-12092	−0.001722069	0.02377249	0.9440305
UIA	7-alpha-hydroxy-3-oxo-4-cholestenoate (7-Hoca)	−0.1928192	0.148959	0.324819

## Discussion

We employed available GWAS data and conducted a two-sample MR analysis to examine the causal associations between blood metabolites and IAs. Potential confounding factors were thoroughly addressed through a comprehensive sensitivity analysis, thereby enhancing the reliability and validity of the findings. Notably, this study represents a pioneering attempt to integrate metabolomic and genomic approaches to investigate the causal relationships between blood metabolites and IAs.

In a study of IAs, Tomoki Hashimoto and colleagues highlighted the promising therapeutic implications of dietary daidzein and its metabolite, equol, in averting the occurrence of IAs and the associated SAHs (23). Additionally, Duan et al. conducted a pseudotargeted metabolomics investigation, systematically elucidating the metabolic profiles and associated pathways involved in the progression of IAs. These findings suggest that blood metabolites may be a potential target for preventing and treating IAs.

The findings of our study reveal significant causal associations between nine specific metabolites and IA. Notably, our analysis highlights the potential involvement of mannose and theobromine, primarily known for their anti-inflammatory properties, in the intricate processes underlying the initiation and progression of IA. These results provide valuable insights into the pathogenesis of this condition and suggest the potential therapeutic relevance of targeting these metabolites in the management of IAs (7).

A substantial body of existing research has consistently demonstrated an association between inflammation and the occurrence and progression of aneurysms (24–27). Experimental studies utilizing macrophage chemotactic protein 1 (MCP1) knockout mice have provided compelling evidence supporting the pivotal role of MCP1 in the initiation of aneurysms. Notably, these studies have shown a significant reduction in macrophage infiltration within high-flow-exposed cerebral arteries of MCP1 knockout mice, resulting in a more than 50% decrease in the initiation and formation of IAs (28). The pivotal role of macrophage infiltration in the initiation of IAs has been consistently supported by multiple studies utilizing various experimental approaches. These studies have employed variations of the classic model of induced IA formation and strategies such as clodronate-induced macrophage depletion (29–31) as well as manipulation of macrophage activation through peroxisome proliferator-activated receptor gamma (PPARγ) modulation (32).

Smooth muscle cells within the IA wall contain receptors for various growth factors secreted by macrophages, including transforming growth factor-β and platelet-derived growth factor-B. These growth factors act as stimulators for smooth muscle cell matrix synthesis and proliferation (33). The continuous activation of macrophages, leading to the secretion of proteases, coupled with their ability to stimulate smooth muscle cells may provide an explanation for the progressive expansion of the aneurysm wall. Following initiation, macrophage activation within the aneurysm wall is further enhanced through an autocrine feedback loop. This loop involves the production of prostaglandin E2 by cyclooxygenase 2 (COX2), which subsequently activates the transcription factor nuclear factor kappa B (NF-κB) in other macrophages. Consequently, the expression of both MCP1 and COX2 is increased in these cells (34, 35). This process induces the recruitment of additional macrophages to the aneurysm wall, thereby triggering NF-κB activation within these cells. Moreover, a comprehensive analysis of gene expression at the genome-wide level in both ruptured and unruptured human IA walls revealed upregulation of multiple genes regulated by NF-κB, specifically in ruptured IA walls. These findings strongly support the implication of macrophage-induced NF-κB activation in the remodeling of established human IAs, similar to that observed in experimental models of induced IA initiation and formation (34, 36, 37).

Mannose, an aldohexose sugar, is abundantly present in various fruits and vegetables, including cranberries, peaches, eggplants, and green beans. Blood concentrations of mannose typically range from 20 to 80 μM, and dietary supplementation can increase levels to over 900 μM, all without observable adverse effects (38). This finding underscores the safety profile of mannose as a naturally occurring sugar. Currently, mannose is widely marketed as a dietary supplement for urinary tract infection management because of its ability to hinder bacterial adherence to the urinary tract (39). Furthermore, recent investigations have highlighted the potential antitumor properties of mannose and its ability to mitigate inflammation in inflammatory bowel disease (40, 41). Nevertheless, the therapeutic potential of mannose in the context of IA remains uncertain and requires further investigation.

Recent research has elucidated the therapeutic effects of mannose in alleviating colitis through its actions on intestinal epithelial cells and macrophages. While the metabolic shift toward increased glycolysis is a well-documented feature of inflammatory macrophages, the precise proinflammatory mechanisms associated with glycolysis remain poorly characterized. Prior research has demonstrated that inhibiting glycolysis can suppress the transcription of inflammatory cytokine genes by restricting the activation of upstream transcription factors, including NF-κB (42, 43). Additionally, evidence supports the notion that mannose exerts a transcription-independent inhibitory effect on tumor necrosis factor (TNF)-α production, which coincides with a reduction in intracellular glycerol-3-phosphate levels induced by mannose. As a result, glyceraldehyde-3-phosphate dehydrogenase is released from glycerol-3-phosphate to bind to TNF-α mRNA (41). The underlying hypothesis of our study posits that mannose acts as an anti-inflammatory agent in the context of IAs, potentially through a similar mechanism. There is no direct evidence that mannose is associated with a reduced risk of IA formation and rupture. However, one study previously found that, in the plasma and aneurysmal tissue of abdominal aortic aneurysm patients, in contrast to CD68 (+) mannose receptor (+) macrophages, CD68 (+) mannose receptor (–) macrophages may contribute to the development of oxidative stress, which is associated with abdominal aortic aneurysm formation in humans (44, 45). This finding provides evidence for a mechanism of action by which mannose attenuates oxidative stress injury by binding to macrophages. Interestingly, oxidative stress injury plays a key role in the development and rupture of IAs (46, 47), and this antioxidant mechanism of mannose may likewise play a similar role in the development and rupture of IAs. Consequently, our MR investigation results revealed a positive association between mannose and a decreased risk of both UIAs and ruptured IAs, signifying its clinical significance. Considering the limited understanding of mannose in the pathophysiological mechanisms of IAs, the identification of mannose as a modulator holds profound implications.

Theobromine, chemically known as 3,7-dimethylxanthine, is a methylxanthine alkaloid found primarily in cocoa beans and dark chocolate and belongs to the same class of plant-derived alkaloids as caffeine and theophylline (48). Extensive research has established that theobromine possesses the capability to impede the adipogenic differentiation process in primary adipose-derived 3T3-L1 mouse cells through suppression of adipogenesis-related factors (49, 50). The interaction between adipocytes and macrophages plays a pivotal role in the inflammatory response *in vivo* (51). Theobromine demonstrated a significant concentration-dependent suppression of lipid accumulation, which was attributed to its ability to decrease the expression of key adipogenic regulators, namely PPARγ and CCAAT/enhancer-binding protein α. Additionally, theobromine exerted antiadipogenic effects by inhibiting AR1 signaling (49, 50). Previous investigations have also provided evidence of the capacity of theobromine to impede the differentiation of preadipocytes into mature adipocytes and reduce the secretion of proinflammatory cytokines, such as MCP1 and interleukin-1β, in the supernatants derived from the interaction between mature adipocytes and macrophages (52). These findings suggest that theobromine has the potential to attenuate proinflammatory compound release, albeit to a partial extent. These findings are in accordance with the outcomes of our MR analysis, indicating a potential role of theobromine in modulating the inflammatory response associated with the development and progression of IAs through inhibition of adipocyte-macrophage interactions. However, the precise underlying mechanism by which theobromine exerts its protective effects against IAs remains unclear and requires further elucidation in future investigations.

An increased risk of IA has been observed in association with elevated androsterone sulfate levels. Androsterone sulfate is a predominant 5-α-reduced androgen metabolite present in serum. Previous investigations have reported markedly increased concentrations of androsterone sulfate in individuals with hyperthyroidism and women exhibiting hirsutism (53, 54). Aberrant endocrine-mediated overproduction of androsterone sulfate has been hypothesized to disrupt the delicate equilibrium between androgens and other hormones, such as estrogen, compromising the protective effects of estrogen against aneurysms. Consequently, the assessment of androsterone sulfate levels holds promise as a potential diagnostic marker, while targeting 5-α-reductase as a therapeutic approach may offer therapeutic benefits for IA management.

Our study identified a suggestive association between the metabolites 7-alpha-hydroxy-3-oxo-4-cholestenoate and N-acetylornithine and UIAs. Nonetheless, the precise underlying mechanisms through which these metabolites contribute to the pathogenesis and progression of unruptured aneurysms will require further investigations of the specific roles and molecular pathways involved.

## Limitations

This study has several limitations. First, it is possible that genetic proxies for blood metabolites may affect the risk of IA through alternate mechanisms, thus challenging the assumptions of MR. Second, the study relied mostly on GWAS summary data from individuals of European ancestry, with limited representation from other racial groups, which may have introduced biases in the findings. Third, although some of the blood metabolites have suggestive causal effects on IA, they may have an underlying non-linear relationship with IA, which may not be resolved in MR studies based on summary-level GWAS data. Finally, the study did not account for the higher incidence of IAs in women than in men, which might affect the results. Future research may benefit from conducting sex-specific MR analyses to address this limitation.

## Conclusion

The results of this study provide evidence for the potential causal effects of four metabolites on IA, two metabolites on aSAH, and three metabolites on UIA, warranting further investigation into the specific roles of blood metabolites in the development of IA.

## Data availability statement

The original contributions presented in the study are included in the article/Supplementary material, further inquiries can be directed to the corresponding authors.

## Ethics statement

Ethical review and approval was not required for the study on human participants in accordance with the local legislation and institutional requirements. Written informed consent from the patients/participants or patients/participants' legal guardian/next of kin was not required to participate in this study in accordance with the national legislation and the institutional requirements.

## Author contributions

JJ: Writing – original draft. SG: Writing – original draft. DW: Writing – review & editing. XC: Writing – review & editing. YT: Formal analysis, Writing – review & editing. JL: Software, Writing – review & editing. WY: Software, Writing – review & editing. TC: Formal analysis, Writing – review & editing. SY: Data curation, Writing – review & editing. HG: Writing – review & editing. YL: Writing – review & editing.
