# Supplementary material for: Causal relationships between human blood metabolites and intracranial aneurysm and aneurysmal subarachnoid hemorrhage: a Mendelian randomization study
Source: Front Neurol. 2023 Dec 14;14:1268138. doi: 10.3389/fneur.2023.1268138 (PMC10755882; doi:10.3389/fneur.2023.1268138)
Supplement: Supplementary file 5 [file Image_1.pdf]

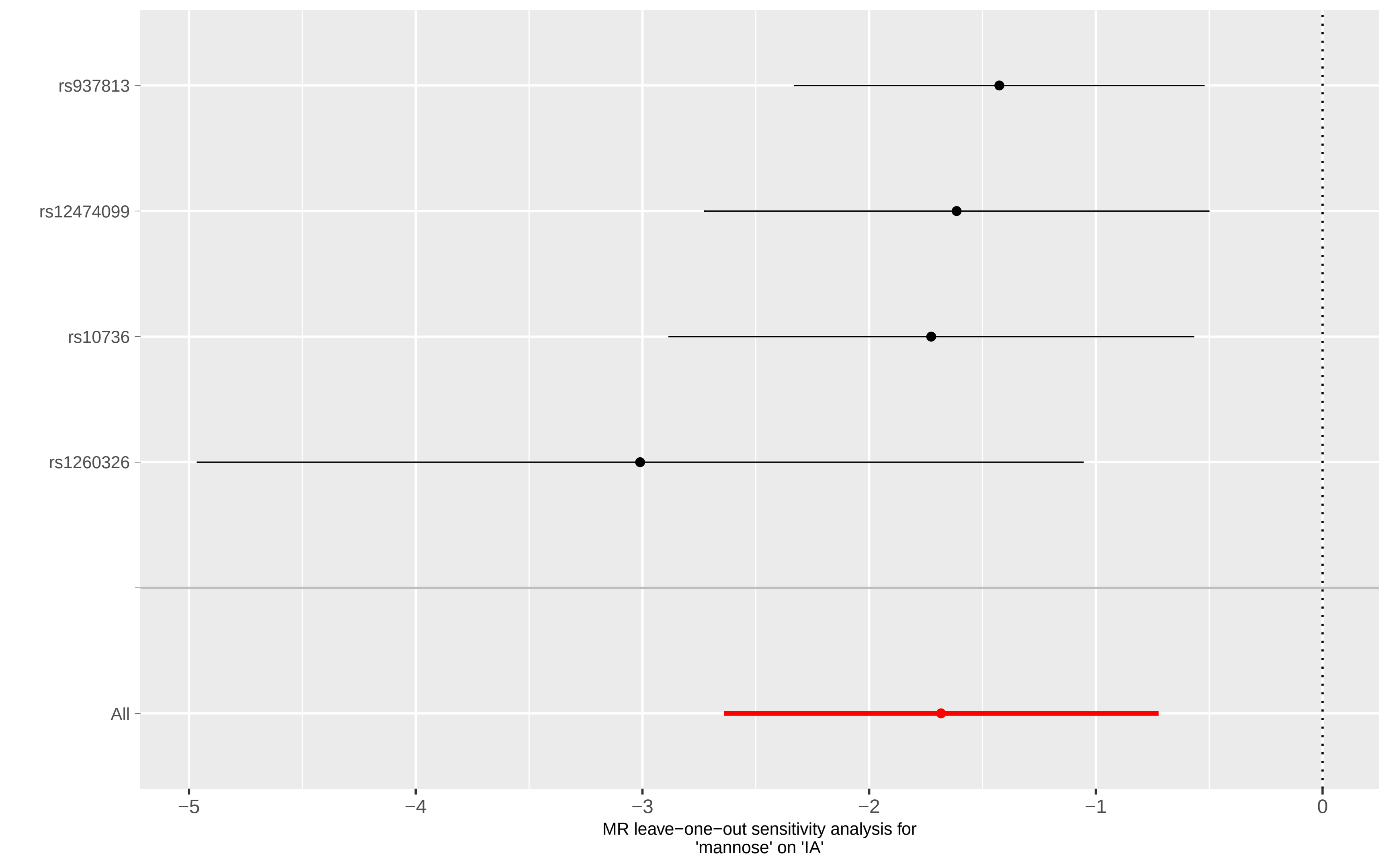

rs937813
rs12474099
rs10736
rs1260326
All
−5
−4
−3
−2
−1
0
MR leave−one−out sensitivity analysis for
'mannose' on 'IA'

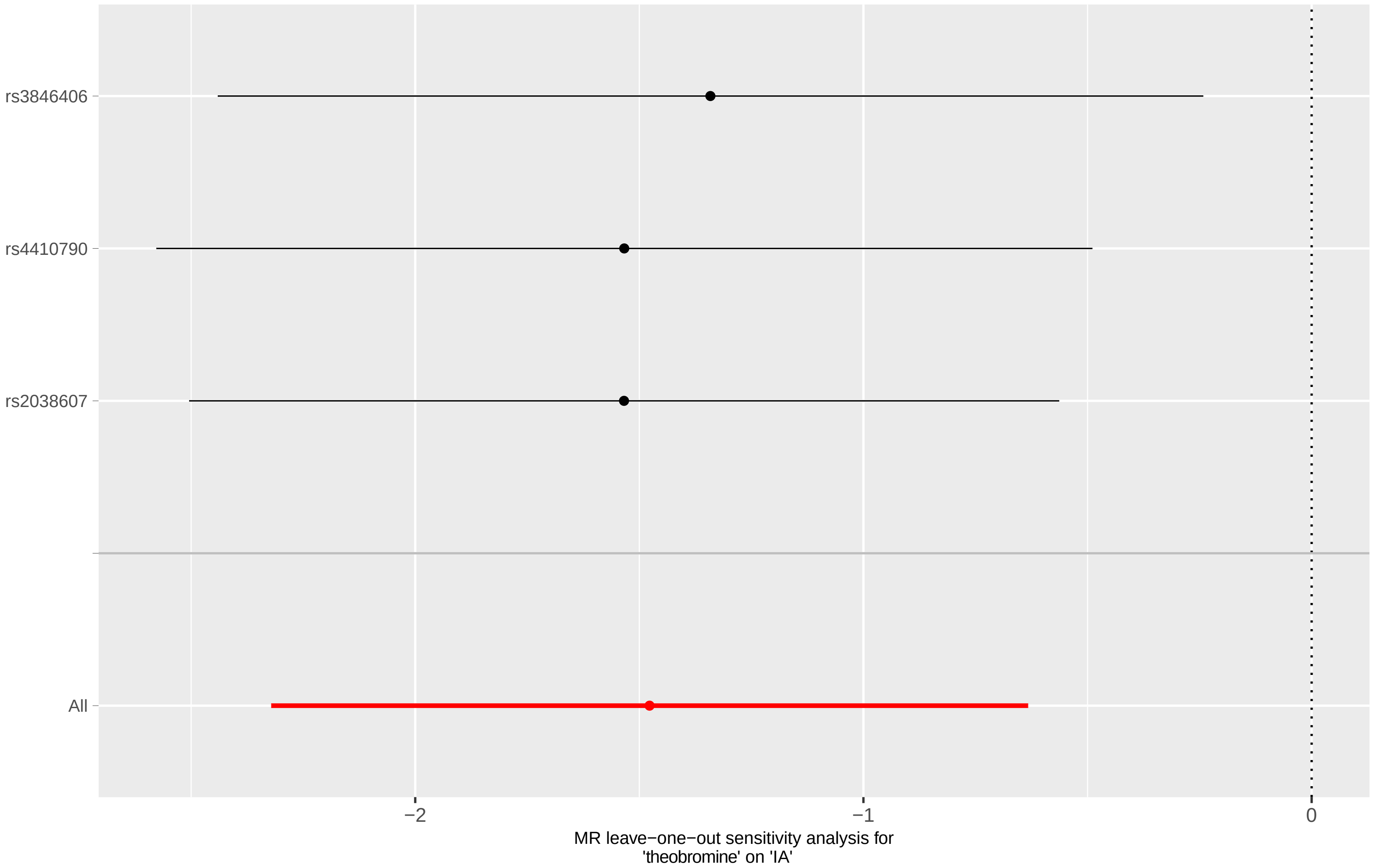
rs3846406
rs4410790
rs2038607
All
−2
−1
0
MR leave−one−out sensitivity analysis for
'theobromine' on 'IA'

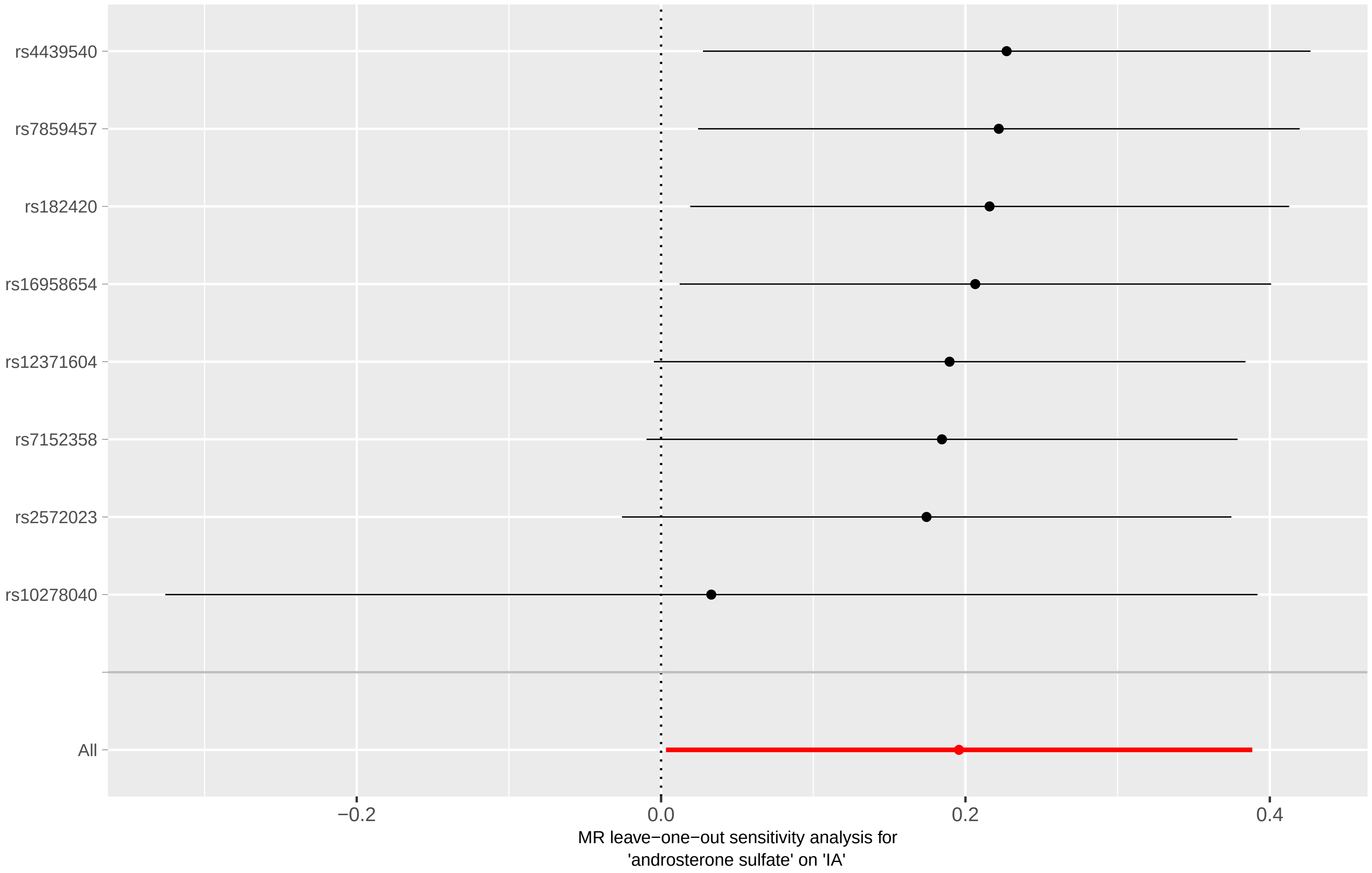

rs4439540
rs7859457
rs182420
rs16958654
rs12371604
rs7152358
rs2572023
rs10278040
All
−0.2
0.0
0.2
0.4
MR leave−one−out sensitivity analysis for
'androsterone sulfate' on 'IA'

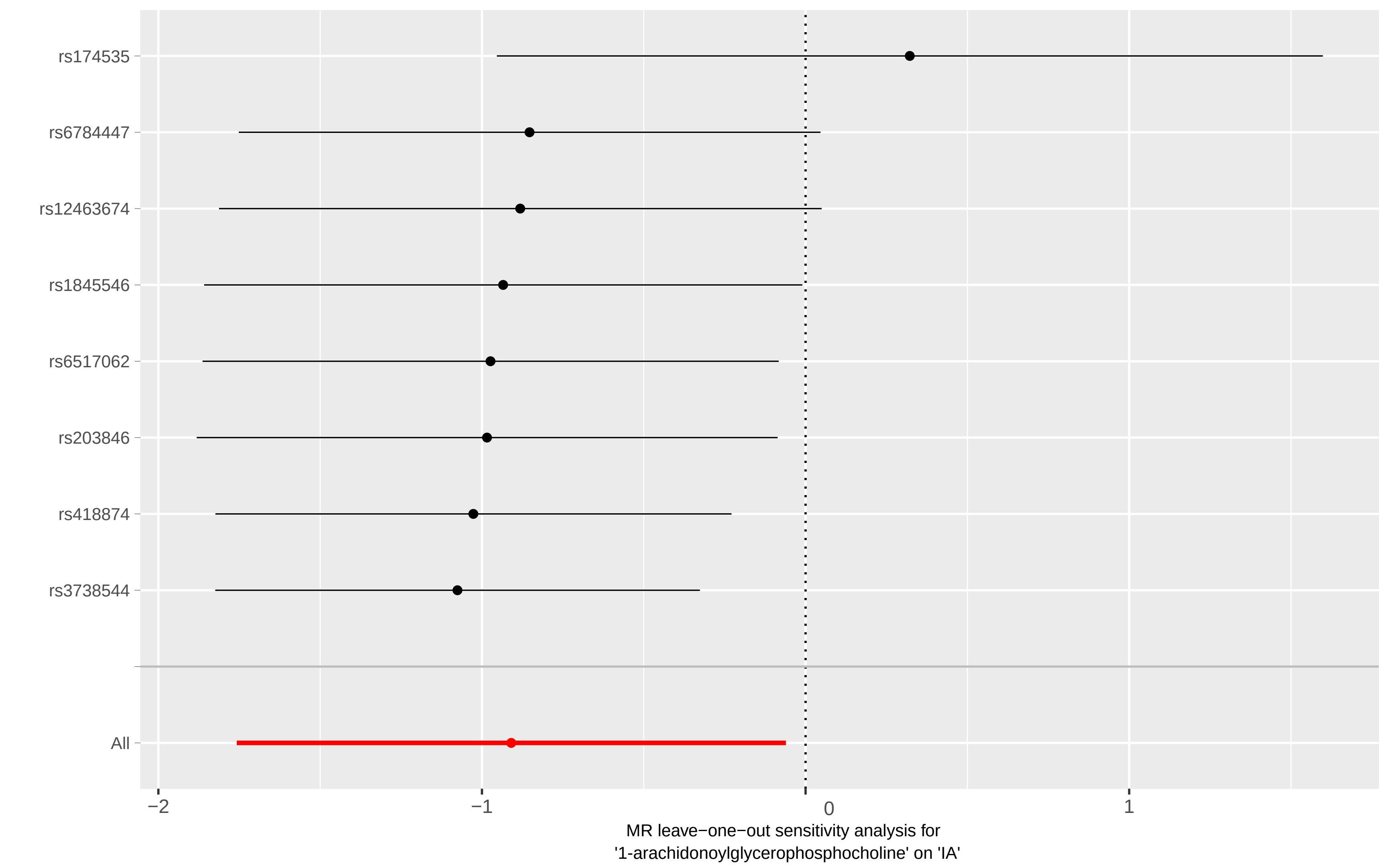

rs174535
rs6784447
rs12463674
rs1845546
rs6517062
rs203846
rs418874
rs3738544
All
−2
−1
0
1
MR leave−one−out sensitivity analysis for
'1-arachidonoylglycerophosphocholine' on 'IA'

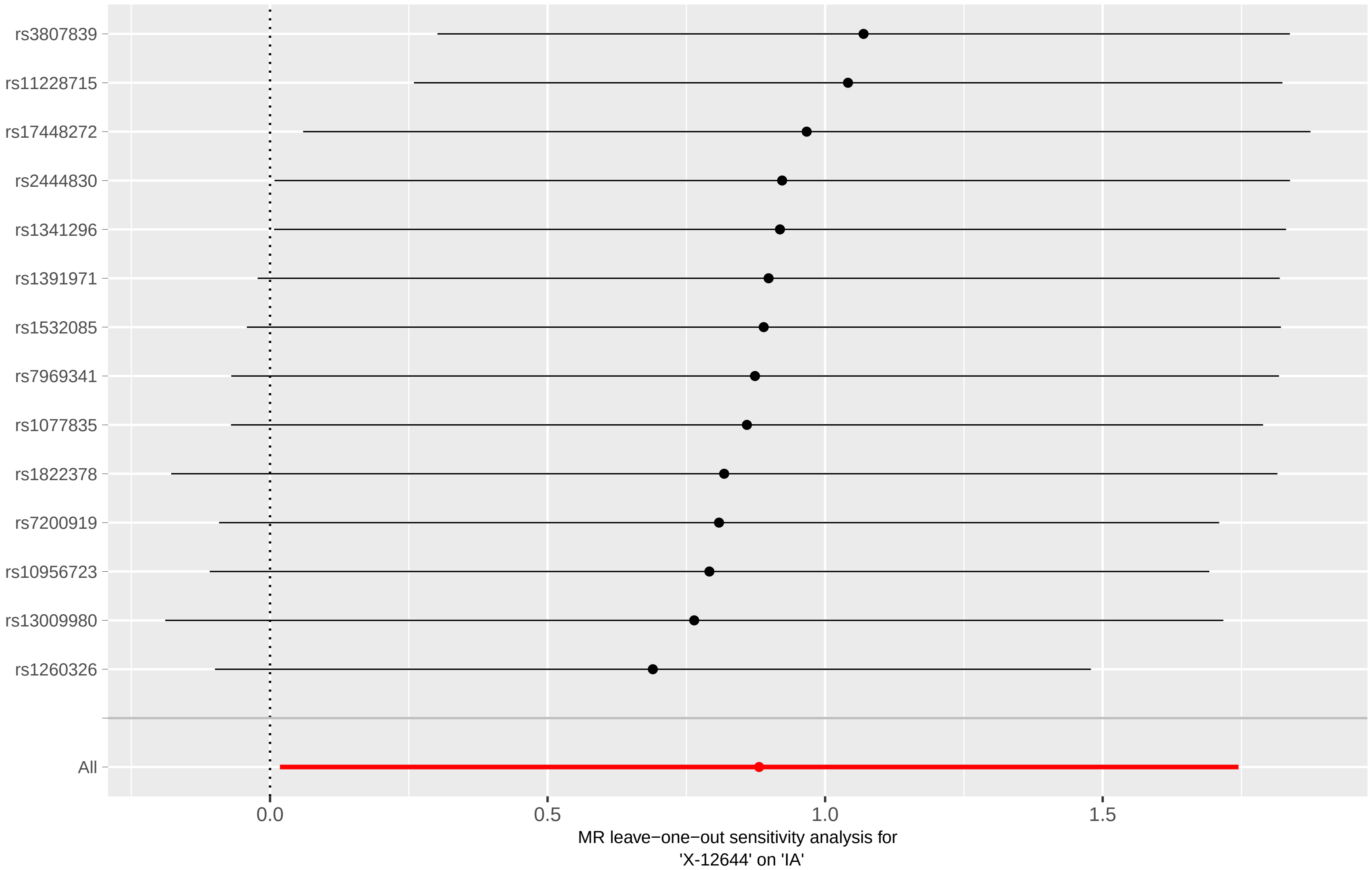

rs3807839
rs11228715
rs17448272
rs2444830
rs1341296
rs1391971
rs1532085
rs7969341
rs1077835
rs1822378
rs7200919
rs10956723
rs13009980
rs1260326
All
0.0
0.5
1.0
1.5
MR leave−one−out sensitivity analysis for
'X-12644' on 'IA'

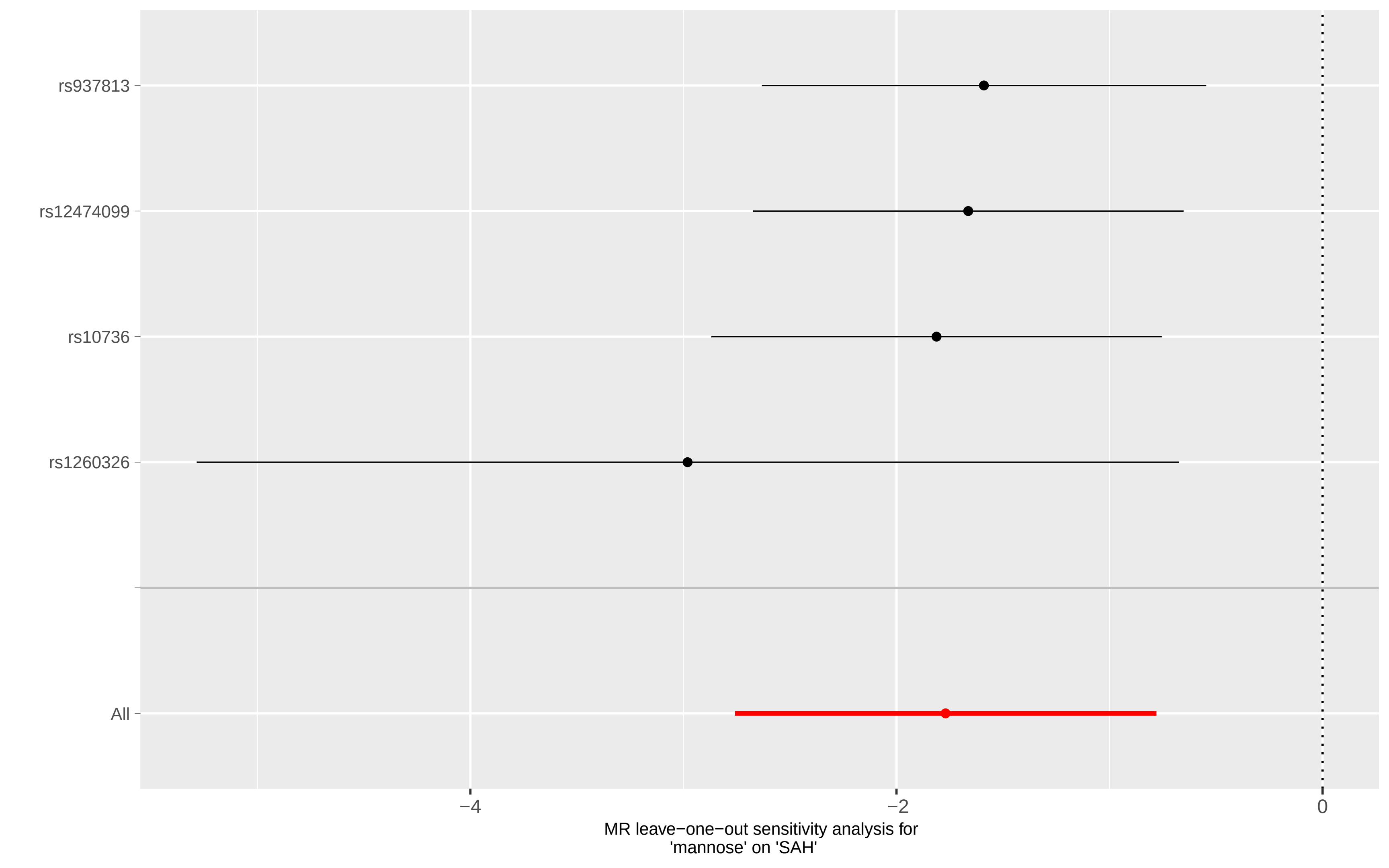

rs937813
rs12474099
rs10736
rs1260326
All
−4
−2
0
MR leave−one−out sensitivity analysis for
'mannose' on 'SAH'

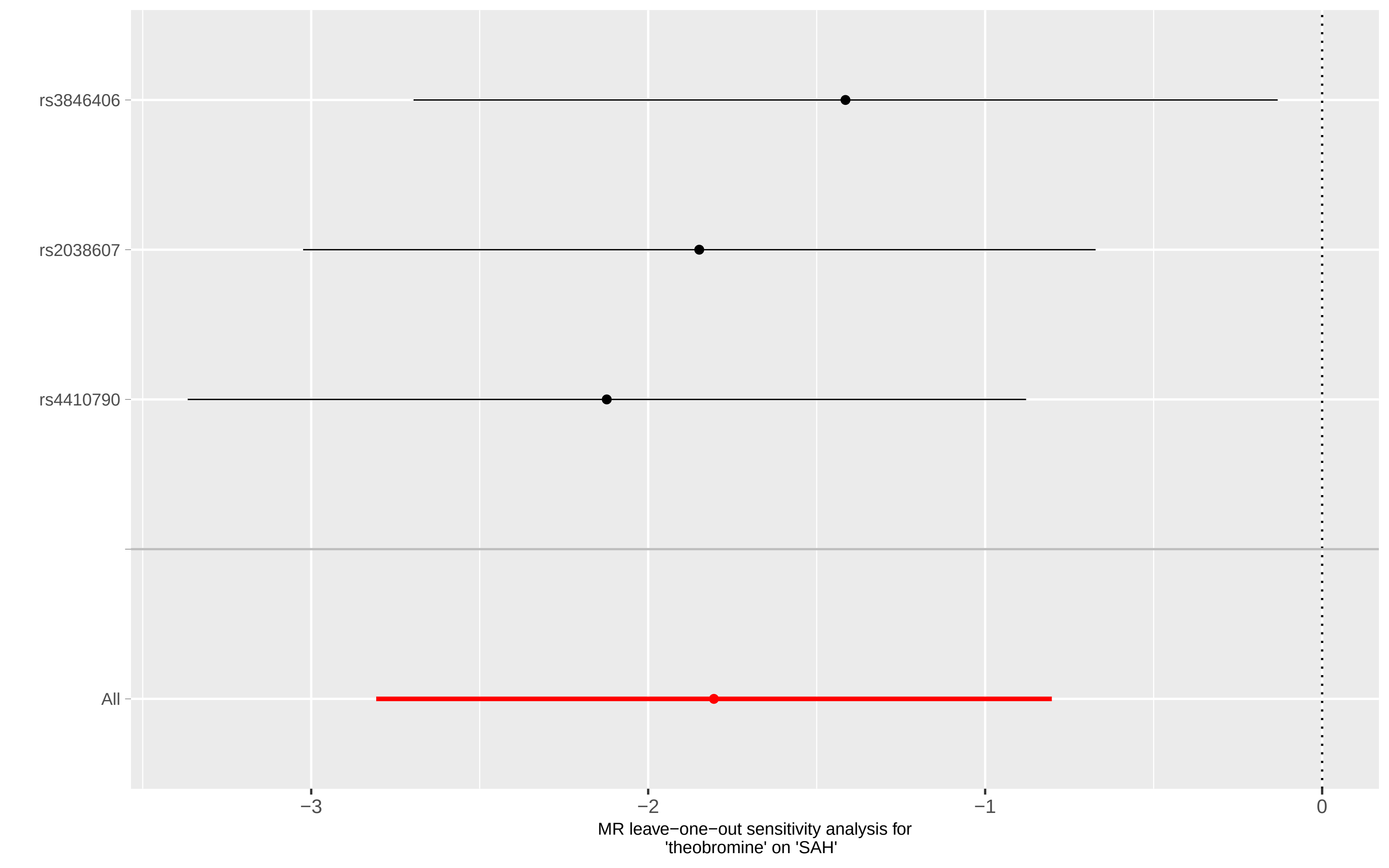
rs3846406
rs2038607
rs4410790
All
−3
−2
−1
0
MR leave−one−out sensitivity analysis for
'theobromine' on 'SAH'

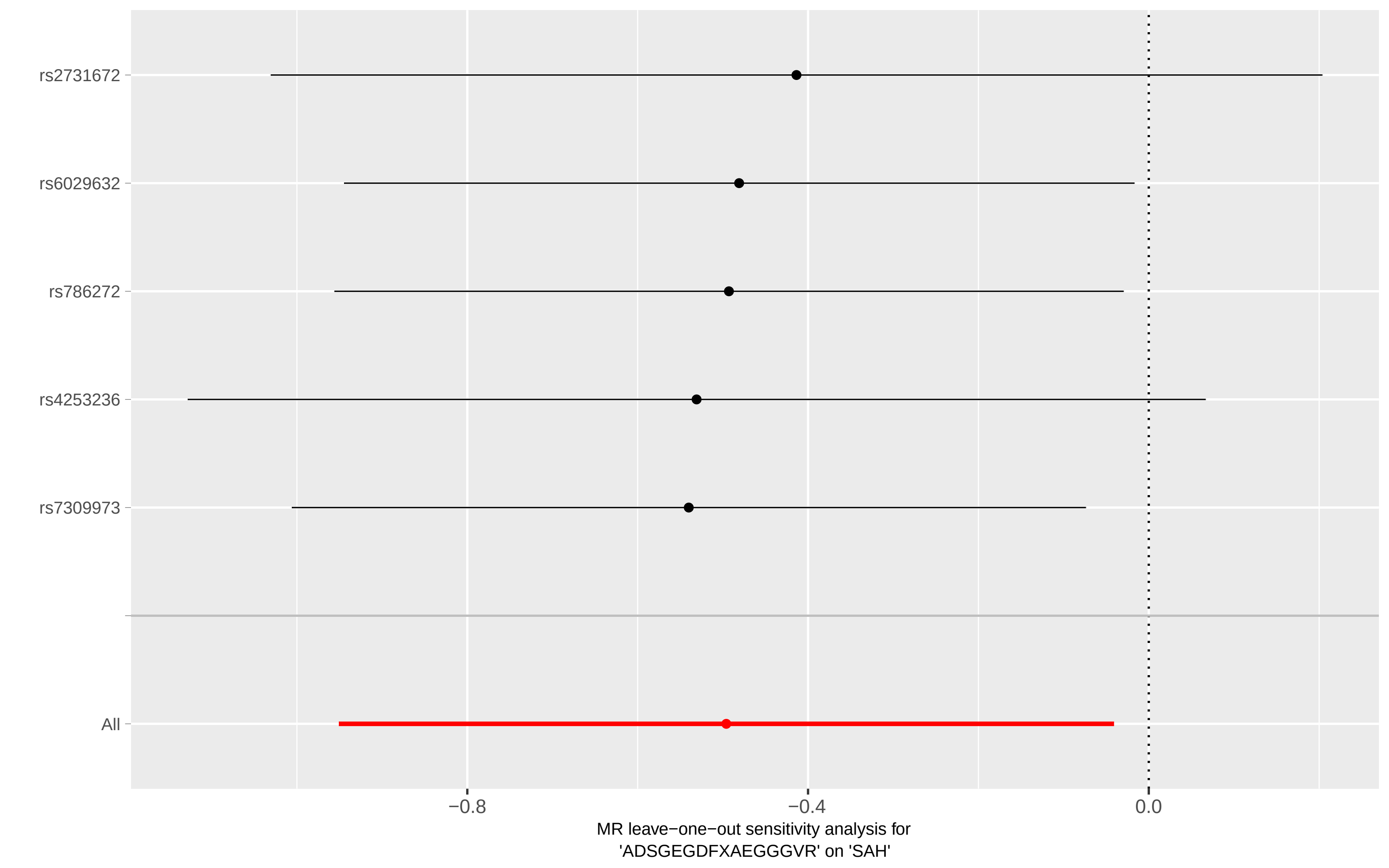
rs2731672
rs6029632
rs786272
rs4253236
rs7309973
All
−0.8
−0.4
0.0
MR leave−one−out sensitivity analysis for
'ADSGEGDFXAEGGGVR' on 'SAH'

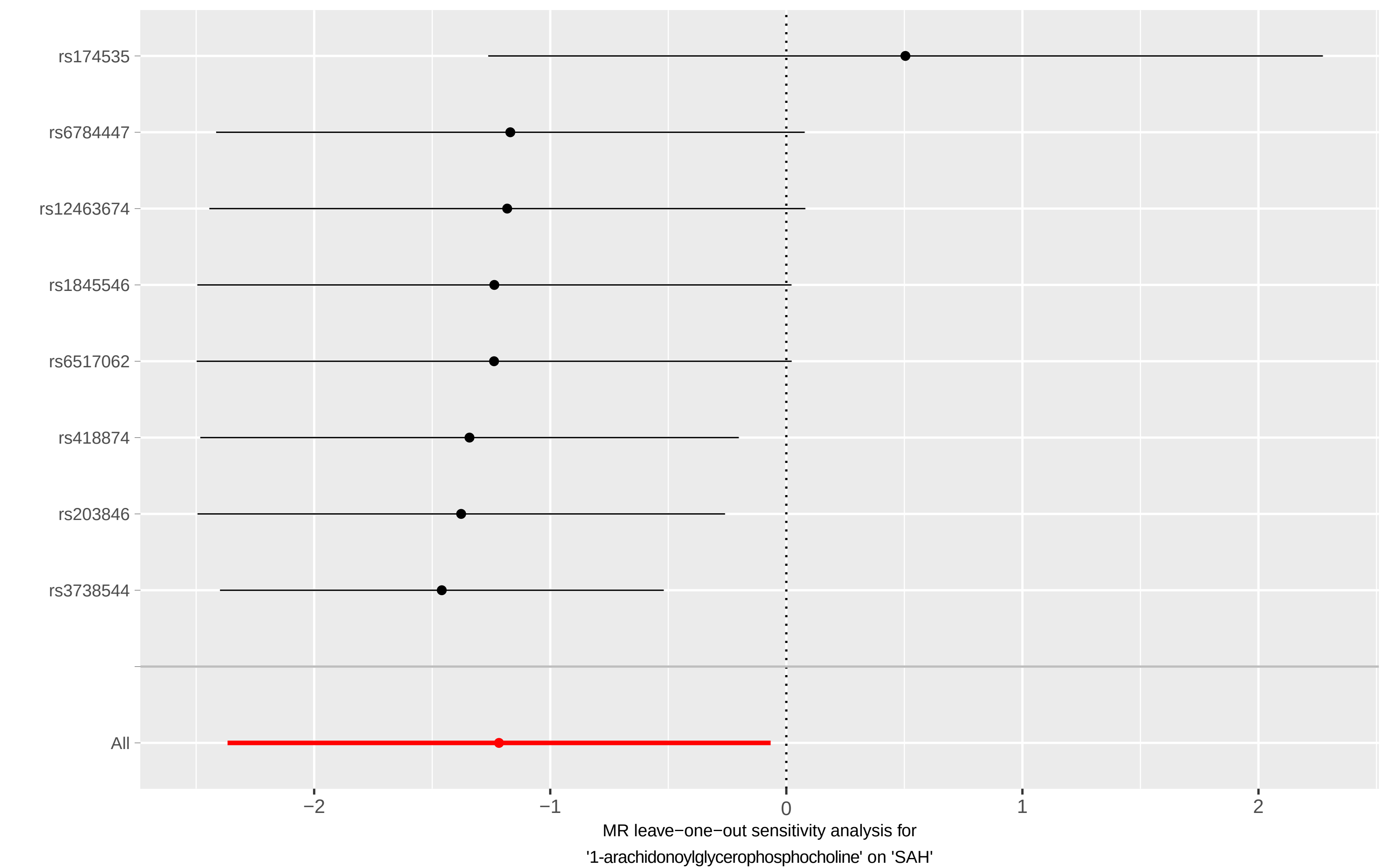
rs174535
rs6784447
rs12463674
rs1845546
rs6517062
rs418874
rs203846
rs3738544
All
−2
−1
0
1
2
MR leave−one−out sensitivity analysis for
'1-arachidonoylglycerophosphocholine' on 'SAH'

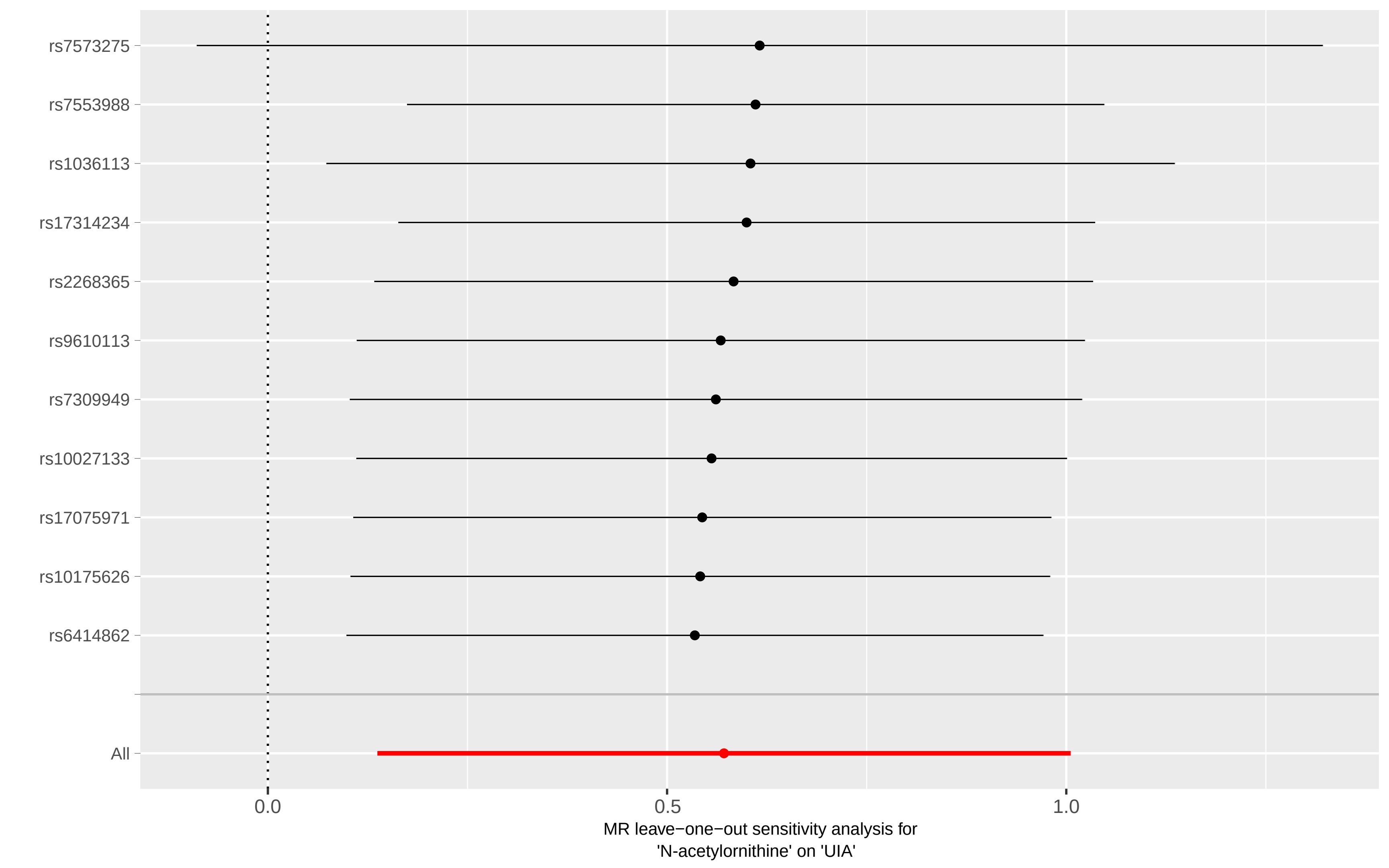
rs7573275
rs7553988
rs1036113
rs17314234
rs2268365
rs9610113
rs7309949
rs10027133
rs17075971
rs10175626
rs6414862
All
0.0
0.5
1.0
MR leave−one−out sensitivity analysis for
'N-acetylornithine' on 'UIA'

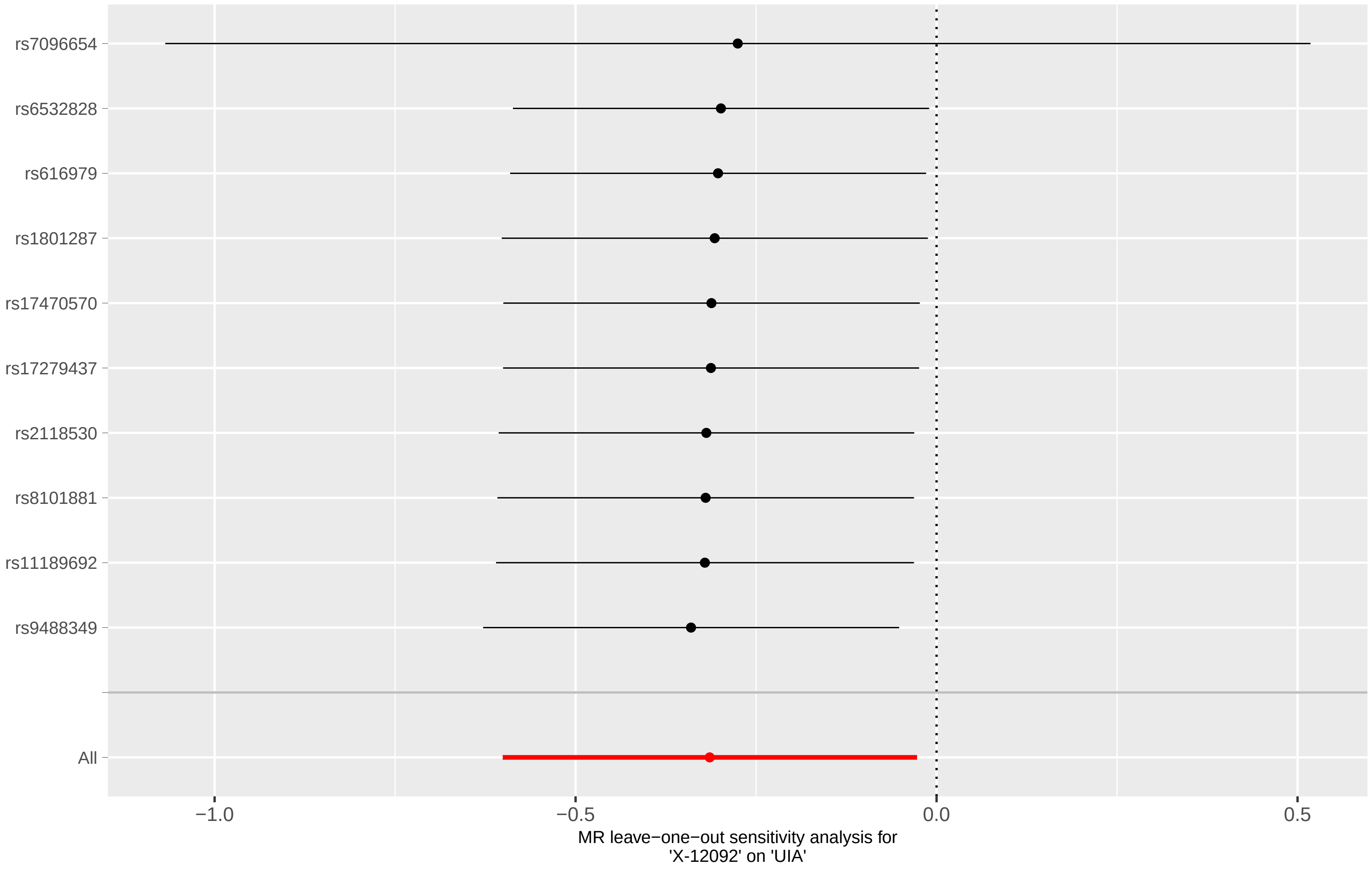
rs7096654
rs6532828
rs616979
rs1801287
rs17470570
rs17279437
rs2118530
rs8101881
rs11189692
rs9488349
All
−1.0
−0.5
0.0
0.5
MR leave−one−out sensitivity analysis for
'X-12092' on 'UIA'

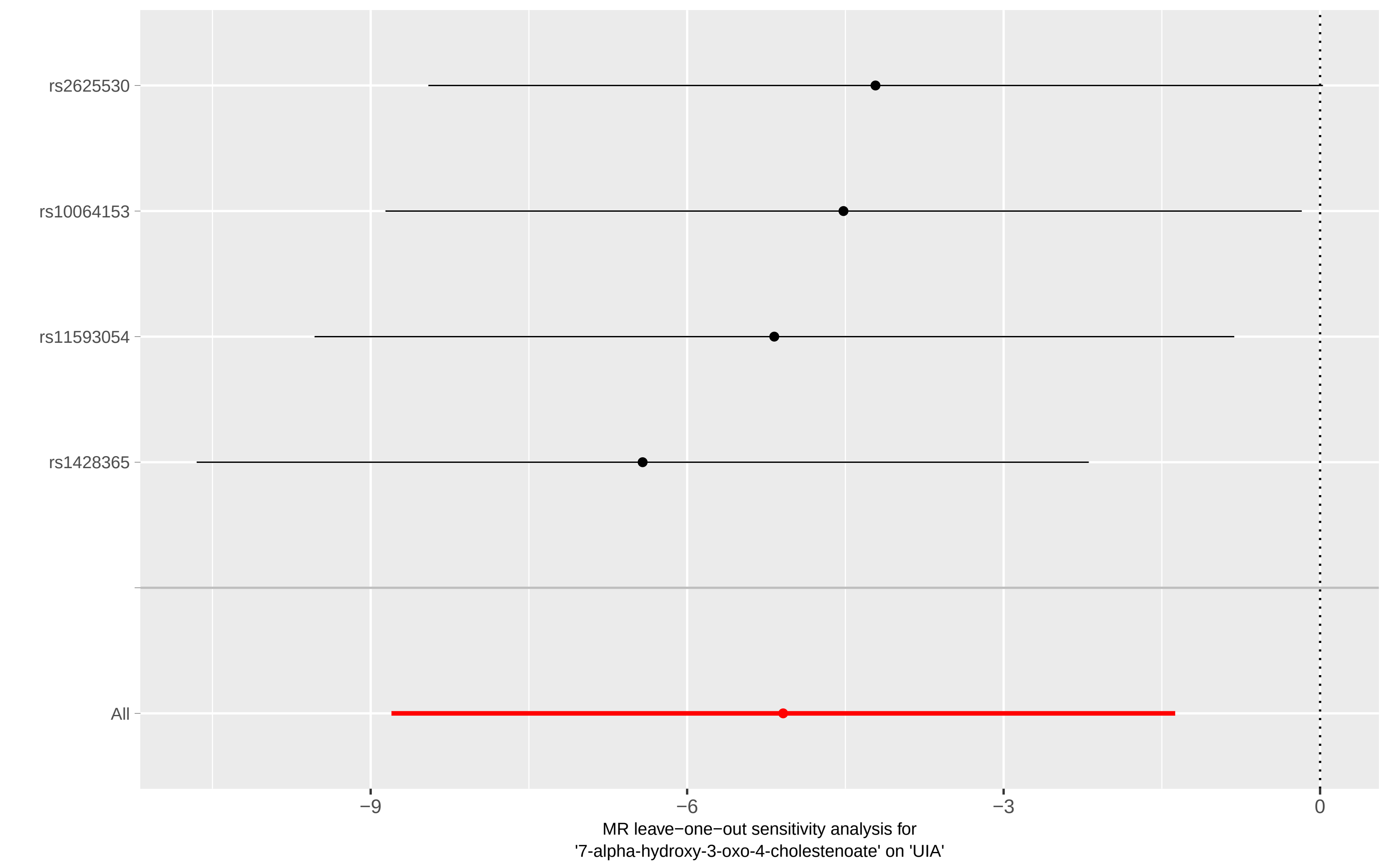
rs2625530
rs10064153
rs11593054
rs1428365
All
−9
−6
−3
0
MR leave−one−out sensitivity analysis for
'7-alpha-hydroxy-3-oxo-4-cholestenoate' on 'UIA'
